# Molecular Partners of Voltage-Gated Calcium Channel β and α_2_δ Auxiliary Subunits: Roles in Channel Complex Regulation and Beyond

**DOI:** 10.1007/s00232-026-00371-w

**Published:** 2026-03-16

**Authors:** Alejandra Corzo-López, Margarita Leyva-Leyva, Ricardo González-Ramírez, Alejandro Sandoval, Ricardo Felix

**Affiliations:** 1 School of Medicine FES Iztacala, National Autonomous University of Mexico (UNAM), Tlalnepantla, Mexico; 2 Department of Molecular Biology and Histocompatibility, Dr. Manuel Gea González General Hospital, Mexico City, Mexico; 3 Department of Cell Biology, Centre for Research and Advanced Studies (Cinvestav), Mexico City, Mexico; 4 Departamento de Biología Celular Cinvestav, Avenida IPN #2508 Colonia Zacatenco Ciudad de México, 07360 CP Mexico City, México

**Keywords:** Calcium channels, Ca_V_β subunit, Ca_V_α_2_δ subunit, Ca_V_ channel regulation

## Abstract

Research on the auxiliary subunits β (Ca_V_β) and α_2_δ (Ca_V_α_2_δ) of voltage-gated calcium channels has gained increasing interest as novel and unexpected functional interactions for these proteins are discovered. Beyond their classic role as regulators of the channel complex, these subunits participate in the spatiotemporal fine-tuning of the channels and in diverse cellular processes. Currently, multiple studies are investigating the interactions between Ca_V_β and Ca_V_α_2_δ with other proteins outside the channel complex, and how these associations affect relevant cellular functions such as cell growth, differentiation, and gene expression beyond their effects on channel activity. The auxiliary subunits have also been observed to associate with components involved in calcium channel biogenesis, a process independent of the direct modulation of channel activity. Furthermore, their involvement in the intracellular transport of other channels and receptors, as well as in nuclear signaling, has been demonstrated. The expression of different variants or isoforms may fulfill specific functions in diverse tissues and developmental stages, even outside the channel complex. Undoubtedly, the study of these proteins as scaffolding or regulatory molecules for other proteins has significantly enriched our understanding of their influence on cell signaling and excitability.

**Graphical Abstract:**

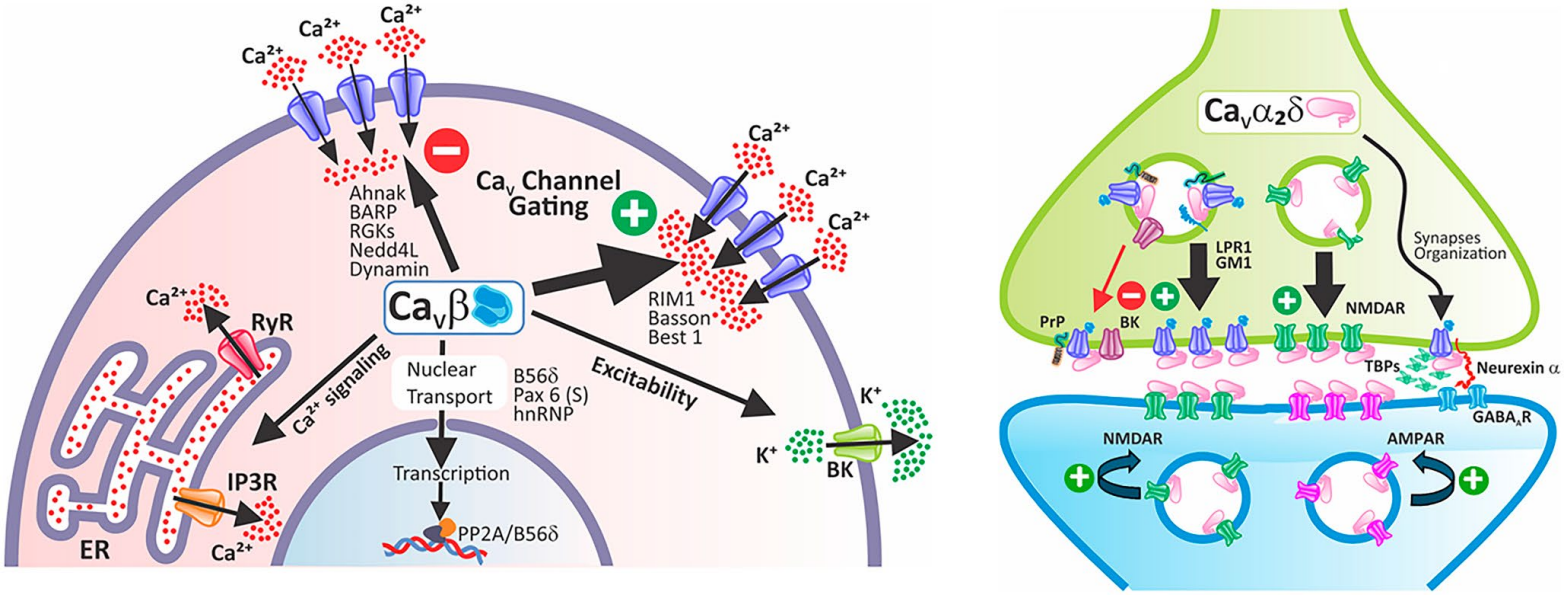

**Supplementary Information:**

The online version contains supplementary material available at 10.1007/s00232-026-00371-w.

## Introduction

Calcium is a key second messenger that controls a wide range of cellular processes, such as cell signaling, enzyme function, excitation-contraction coupling, neurotransmitter release, gene expression, cell movement, growth, and programmed cell death (Zamponi et al. 2015). Due to its relevance, intracellular calcium levels are precisely regulated by the coordinated action of pumps and exchangers in the plasma membrane that extrude excess cytosolic free calcium, and various intracellular compartments that recapture and store it (Berridge et al. 2003). Among the diverse mechanisms that regulate calcium entry into the cytosol in excitable cells are voltage-gated calcium (Ca_V_) channels, of which there are two classes, low and high threshold activation channels, LVA and HVA, respectively (Zamponi et al. 2015).

Unlike LVA channels, which may function as monomers of the pore-forming (Ca_V_α_1_) subunit of the channels, HVA channels are heteromultimeric complexes that have auxiliary subunits (Ca_V_β, Ca_V_α_2_δ, and, in some cases, Ca_V_γ), which regulate the biophysical properties of the channels, as well as their intracellular transit, their stability in the plasma membrane, and their proteasomal degradation (Zamponi et al. 2015). HVA channels are classified into two main subfamilies: the first is the Ca_V_1 channels, also known as L-type channels, which comprise four members (Ca_V_1.1 to Ca_V_1.4). The second is the Ca_V_2 subfamily, comprising three members, Ca_V_2.1 (P/Q-type), Ca_V_2.2 (N-type), and Ca_V_2.3 (R-type). Low-voltage-activated (LVA) channels comprise three members, Ca_V_3.1 to Ca_V_3.3, also known as T-type channels (Suppl. Table 1).

Numerous excellent review articles cover the topics discussed here; key examples appear in the references. For deeper insight into any area, we encourage readers to consult them. To enhance readability, we’ve also provided a complete, alphabetical list of abbreviations as supplemental material.

## Molecular Structure of Ca_V_ Channels

**Fig. 1:**
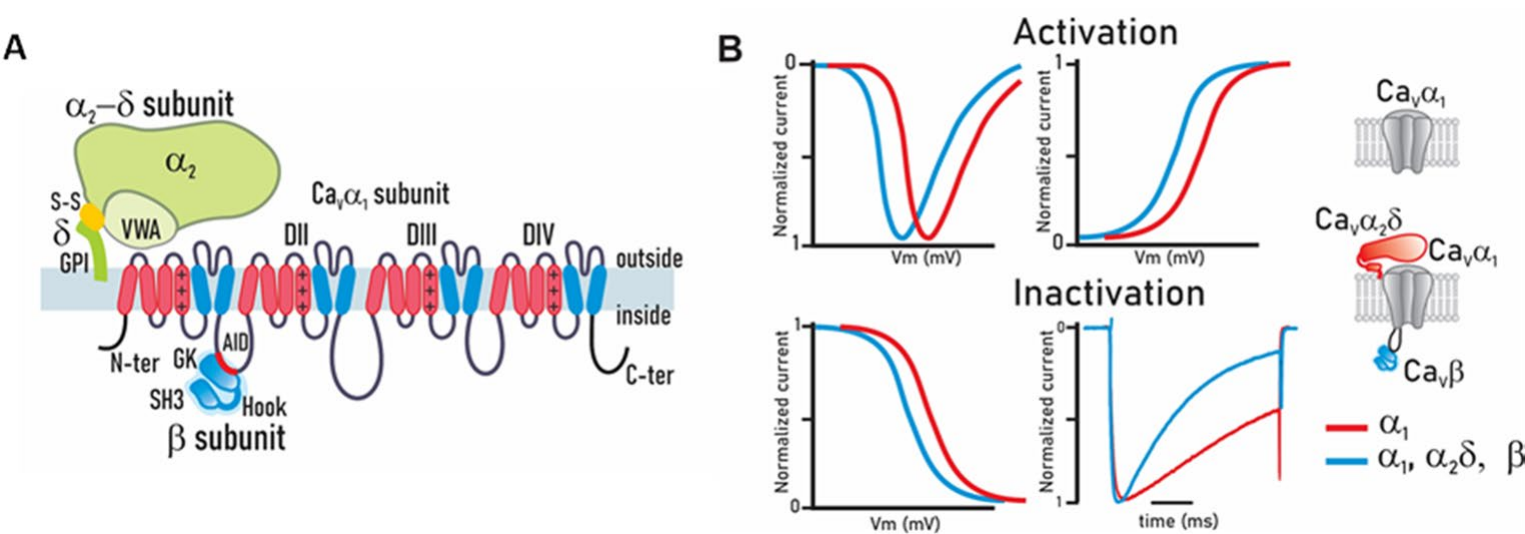
Structure and function of voltage-gated calcium channel subunits of the Ca_V_1 and Ca_V_2 subfamilies. (**A**) The main subunit, called Ca_V_α_1_, is a protein weighing > 200 kDa and is made of four similar regions (domains DI-DIV). Each domain contains six segments that cross the cell membrane, called segments S1 to S6. The Ca_V_α_1_ subunit forms the calcium-permeable pore, with each domain’s S4 segment serving as the voltage sensor that detects membrane potential changes. Membrane potential changes induce channel opening, with auxiliary Ca_V_β and Ca_V_α_2_δ subunits modulating function and localization—Ca_V_β intracellularly and Ca_V_α_2_δ extracellularly. Ca_V_β localizes intracellularly, whereas Ca_V_α_2_δ resides extracellularly. (**B**) The auxiliary subunits increase the current amplitude through calcium channels by increasing channel open probability and the functional expression of the channel complex at the plasma membrane. These proteins shift both activation and steady-state inactivation curves to more negative potentials while accelerating inactivation kinetics, thereby facilitating low-voltage channel opening and underscoring auxiliary subunits’ critical role. The right inserts show channel composition and subunits

The pore-forming subunit (Ca_V_α_1_) consists of four repeated homologous domains (I to IV), each with six α-helix transmembrane segments designated S1 to S6, which together form a highly selective pore for calcium ions and a complex system for voltage sensing and channel regulation. It is a large protein (~ 170–250 kDa) essential for channel function. Structurally, each domain contributes two functional components. The first four transmembrane segments (S1–S4) constitute the voltage-sensing domain. Notably, the S4 segment contains positively charged amino acids (aa)—predominantly arginines and occasionally lysines—that are essential for sensing membrane potential changes, thereby gating the channel open or closed. The second functional component is formed by the transmembrane segments S5 and S6 of each repeated domain, which constitute the pore region. Through the hydrophobic loops connecting them, they help create the selectivity filter that determines which ions can pass through the pore (Fig. 1A; Zamponi et al. 2015).

Furthermore, as discussed below, the Ca_V_α_1_ subunit has intracellular regions that serve as regulatory sites, including sites for interaction with the different Ca_V_β auxiliary subunits and other regulatory proteins such as calmodulin. Thus, in addition to being responsible for ion conduction and sensing changes in transmembrane voltage, it is also responsible for many of the interactions with endogenous agents, drugs, and toxins that regulate channel activity (Zamponi et al. 2015).

The Ca_V_β auxiliary subunit is essential for the correct expression in the cell membrane of HVA class Ca_V_ channels. This cytoplasmic protein is encoded by four distinct genes, producing four variants: Ca_V_β_1_ through Ca_V_β_4_. The Ca_V_βsubunits bind to the main Ca_V_α_1_ subunit via two key regions: the AID on the intracellular loop between domains I and II of Ca_V_α_1_, and a 16-aa Beta Interaction Domain (BID) in Ca_V_β (Pragnell et al. 1994; De Waard et al. 1996). It is well known that Ca_V_β subunits interact with the Ca_V_α_1_ subunit to regulate its essential biophysical properties (Fig. 1B) and ensure its correct delivery and localization in the plasma membrane, in addition to preventing its retention in the endoplasmic reticulum (ER) and its possible internalization that would lead to degradation via the ubiquitin proteasome pathway (Colecraft et al. 2002; Yasuda et al. 2004; Altier et al. 2011).

On the other hand, Ca_V_β subunits are now understood as multifunctional proteins due to their interactions with an expanding variety of molecular partners involved in different cellular signaling pathways. These studies have revealed new functions for Ca_V_β beyond its main role in regulating HVA channels (Gandini and Zamponi 2022). For instance, as will be discussed later, research has found Ca_V_β subunits in the cell nucleus, suggesting they might play a role in controlling gene transcription.

Likewise, HVA channels are associated with the Ca_V_α_2_δ ancillary subunit, of which four variants are known, all generated from different genes (Dolphin 2018). Each is translated into a precursor protein that is proteolytically processed to produce an extracellular δ region associated with the cell membrane through a glycophosphatidylinositol (GPI) anchor, and an α_2_ region that is highly glycosylated and contains the von Willebrand factor A (VWF-A), MIDAS, and Cache functional domains (Dolphin 2018). A disulfide bond holds the α_2_ and δ regions together (Calderón-Rivera et al. 2012). Likewise, it is known that the Ca_V_α_2_δ subunit modulates the electrophysiological properties of the Ca_V_ channel complex, primarily increasing current density and accelerating inactivation kinetics (Fig. 1B; Felix et al. 1997; Andrade et al. 2009). In addition, its presence may influence the pharmacological features of the channel complex (Mould et al. 2004; Andrade et al. 2009). Lastly, as an extracellular protein, Ca_V_α_2_δ may perform cellular functions outside the channels as a result of its interaction with proteins of the extracellular matrix involved in synaptic transmission and synaptogenesis, such as neurexins and thrombospondins (TSPs), as we shall discuss later (Eroglu et al. 2009; Zamponi et al. 2015; Schöpf et al. 2021).

## The Ca_V_β Auxiliary Subunit: Structure and Function

**Fig. 2:**
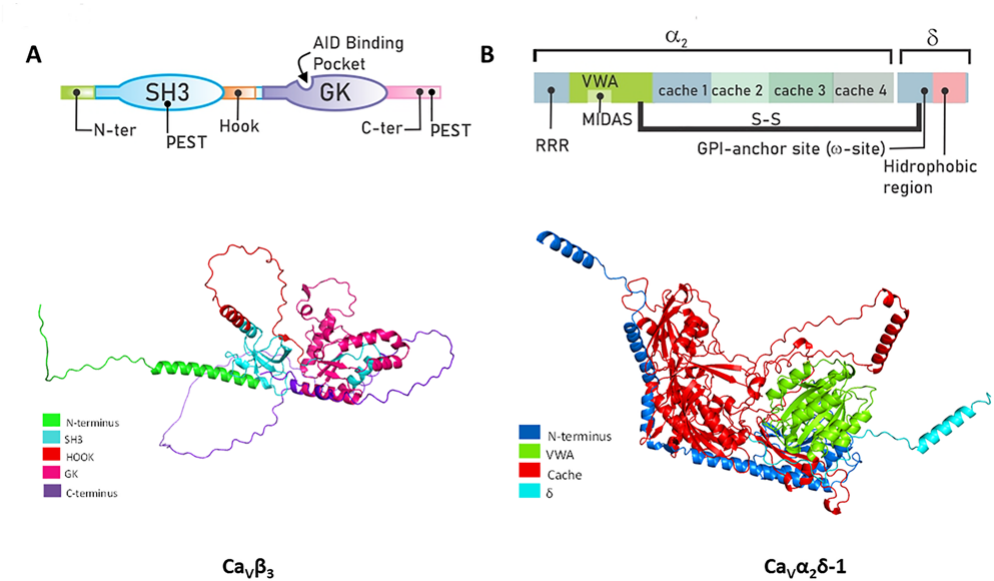
Structural analysis of the calcium channel Ca_V_β and Ca_V_α_2_δ auxiliary subunits. (**A**) The Ca_V_β subunit contains functional domains (upper panel): an enzymatically inactive guanylate kinase (GK) domain that supports channel structure/function; an SH3 domain that binds intracellular proteins to regulate activity; and a HOOK region linking GK and SH3. Ca_V_β also binds the Ca_V_α_1_ subunit via the AID region and has two PEST-like sequences that target it for calpain degradation. The lower panel shows the structure of the full Ca_V_β_3_ protein predicted using the AlphaFold Protein Structure Database (Jumper et al. 2021; Varadi et al. 2022; 2024). To illustrate the protein structure, the Ca_V_β3 isoform was used, which yielded the highest average confidence score (Local Distance Difference Test or pLDDT value of 73.78) at the time of analysis. Also, the corresponding Uniport sequences were used for domain characterization. (**B**) The Ca_V_α_2_δ subunit has several key functional domains. The VWF-A domain aids channel trafficking to the membrane via a conserved MIDAS sequence. Cache domains act as ligand-binding sites for protein interactions and GBP binding. It features multiple glycosylation sites for proper trafficking and membrane stability, consists of α_2_ and δ peptides linked by a disulfide bond, and the δ peptide has a GPI anchor for membrane attachment. The lower panel shows the complete structure of the Ca_V_α_2_δ protein, predicted in the same way as for Ca_V_β_3_. To illustrate the protein structure, the Ca_V_α_2_δ−1 isoform was used, which yielded the highest average pLDDT value (86.49). As in the case of Ca_V_β_3_, the corresponding Uniport sequences were used for domain characterization as indicated

The different variants of Ca_V_β share a similar structure composed of five distinct structural domains (Fig. 2A). A conserved tripartite core—SH3, HOOK, and GK-like domains—defines these proteins. High conservation characterizes the SH3 and GK domains across isoforms, in contrast to the divergent HOOK and terminal domains (Chen et al. 2004, 2009; Opatowsky et al. 2004; Van Petegem et al. 2004). Furthermore, these domains are subject to alternative splicing and are potential targets for post-translational modifications, primarily phosphorylation and palmitoylation (Buraei and Yang 2010). The GK and SH3 domains of the Ca_V_β subunit are similar to a group of scaffolding proteins called membrane-associated guanylate kinases (MAGUK; McGee et al. 2004). Additionally, the Ca_V_β sequence includes two PEST motifs that mediate protein cleavage by calpain (Sandoval et al. 2006).

All Ca_V_β subunits share the same domain structure, but the catalytic region of the GK domain becomes inactive due to mutations in specific residues (Chen et al. 2004; Opatowsky et al. 2004; Van Petegem et al. 2004). In contrast, the SH3 domain, which comprises ~ 60 aa that form five-stranded antiparallel β-sheets (Fig. 2A, lower panel; Yu et al. 1994), can interact with proteins containing proline-rich motifs (PxxP). Notably, the last two β-strands are separated by the HOOK region in the SH3 domain of the Ca_V_β subunit. Structural analysis of the protein reveals that part of the HOOK region obstructs the PxxP binding site (Fig. 2A, lower panel; Chen et al. 2004; Opatowsky et al. 2004; Van Petegem et al. 2004). Nevertheless, this binding site may become accessible through a conformational change when Ca_V_β associates with the Ca_V_α_1_ subunit (Chen et al. 2004; Opatowsky et al. 2004).

The interaction between Ca_V_α_1_ and Ca_V_β helps stabilize the channel complex and aids in its transport from the ER. The binding of Ca_V_β to the AID domain may mask Ca_V_α_1_ retention signals (Bichet et al. 2000; Fang and Colecraft 2011). The channel, therefore, no longer remains in the ER and begins its transit to the cell surface. This increases the channel density at the cell surface by enabling the channel complex to exit the ER and move via the secretory pathway to the cell membrane. Additionally, this association blocks the channel complex from being recognized by the ER-associated degradation pathway. Ca_V_β helps keep the Ca_V_α_1_ subunit stable at the cell membrane by preventing it from being marked for degradation (Opatowsky et al. 2004; Chen et al. 2004; Altier et al. 2011; Waithe et al. 2011).

All four Ca_V_β variants share 40–50% identity in their core sequences, which supports their common roles in stabilizing voltage-gated calcium channels and facilitating their trafficking to the plasma membrane. The guanylate kinase (GK) domain and critical interaction motifs display particularly high conservation, yielding uniform impacts such as increased peak Ca²⁺ currents and altered voltage-activation curves. N- and C-terminal regions have lower identity: Ca_V_β_1_ has a unique N-terminal module, while Ca_V_β_2_ features a variable HOOK loop (residues 275–295) that is absent or changed in others, influencing binding and membrane association (Morgenstern 2019; 2022).

Recent research by Minor and colleagues provides a new perspective on Ca_V_ channel assembly, emphasizing specific molecular interactions with Ca_V_β subunits (Chen et al. 2023). These authors determined that the Ca_V_α_1_ and Ca_V_β subunits, along with a chaperone known as the ER membrane protein complex (EMC), form an intermediate molecular complex during channel assembly. Interaction with this chaperone causes specific structural shifts in the channel, preparing it for the subsequent addition of the Ca_V_α_2_δ subunit, which finalizes the channel complex assembly. During this process, key areas of Ca_V_α_1_ reorganize, and a divalent ion helps coordinate the subunits. Likewise, the association of the EMC and the Ca_V_α_2_δ auxiliary subunit is mutually exclusive, suggesting that the EMC acts as an intermediary that stabilizes the channel while it reaches maturation and completes assembly (Chen et al. 2023).

This novel intermediary complex forms specifically when the Ca_V_β subunit binds directly to the EMC. Indeed, structural studies have revealed that several conserved residues within the Ca_V_β sequence interact with specific sites on the EMC, mainly in the EMC8 and EMC2 regions. Ca_V_β_3_ binding stabilizes the nascent Ca_V_1.2α_1_/Ca_V_β_3_ complex prior to Ca_V_α2δ incorporation, as shown by mutagenesis. Ablating these sites reduces interaction with the endoplasmic reticulum membrane complex (EMC) and compromises functional expression, confirming the interaction’s key role in channel biogenesis (Chen et al. 2023).

## Non-canonical Interactions of the Ca_V_β Subunits

Ca_V_β subunits regulate Ca_V_ channel biophysics and trafficking (Fig. 1B; Yasuda et al. 2004; Buraei and Yang 2010) while mediating channel-independent protein-protein interactions that modulate diverse cellular processes (Buraei and Yang 2010; Rima et al. 2016).In recent years, the list of proteins that interact with the Ca_V_β subunits has expanded, and the functional implications of these molecular associations have begun to be described. Its ability to form protein complexes comes from its unique structure and the numerous variant forms that have been identified (Buraei and Yang 2010; Rima et al. 2016).

**Fig. 3:**
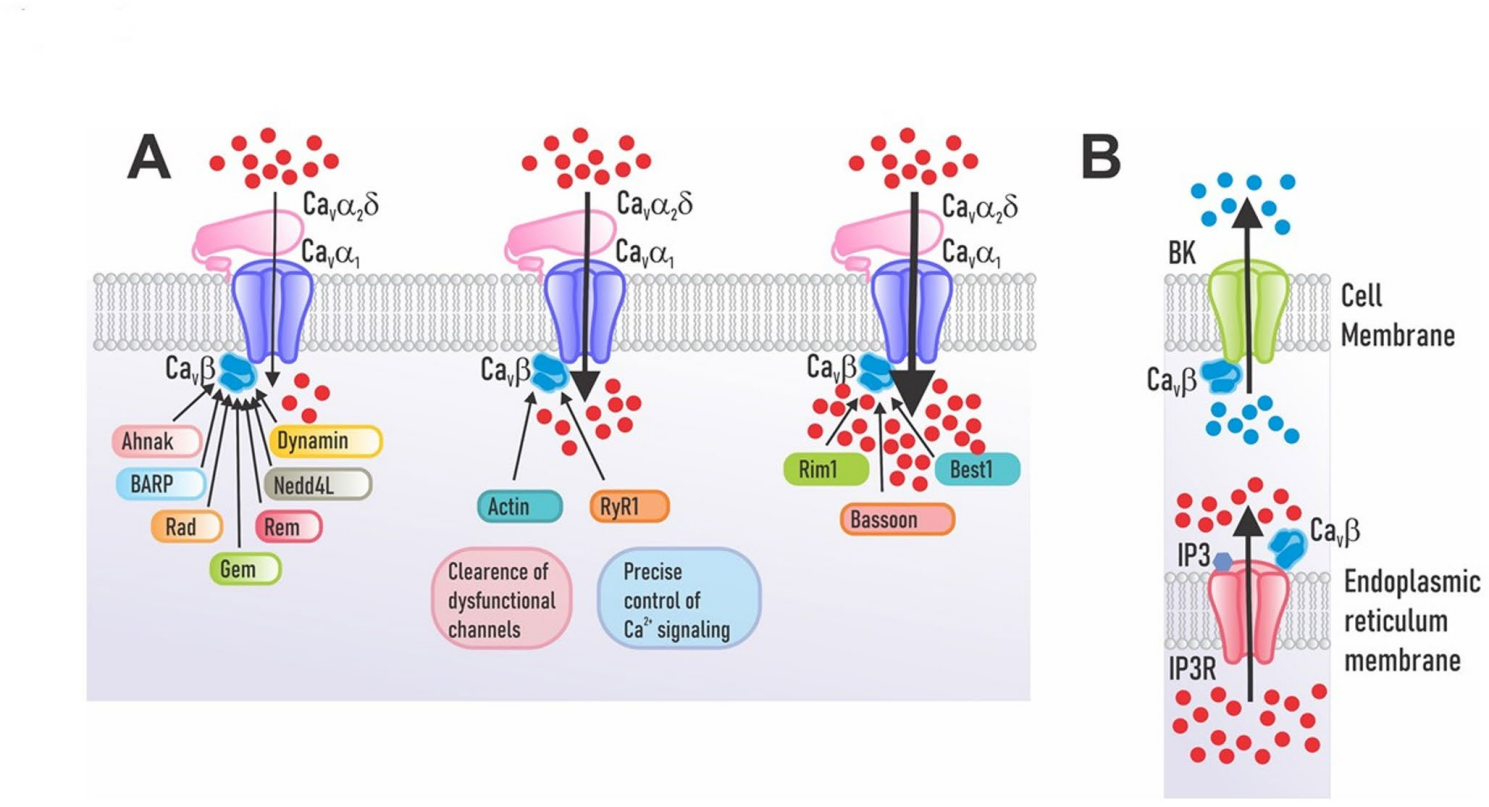
Expanding the view of Ca_V_β subunit interactions outside the calcium channel complex. (**A**) Ca_V_β directly interacts with various intracellular proteins that regulate Ca_V_ channel function. Key ones include: RGK family GTP-binding proteins, the strongest inhibitors; Ahnak, a scaffolding protein for cell structure and Ca²⁺ signaling; BARP, a Ca_V_-linked regulator not in a large family; Dynamin, a GTPase from the dynamin superfamily that remodels membranes in endocytosis; and NEDD4L, an E3 ubiquitin ligase from the NEDD4 family that tags proteins for degradation. (**B**) Ca_V_β can associate with BK channels to control cellular excitability and with IP3R to help control intracellular calcium signaling

Ca_V_β subunits contribute to presynaptic organization by binding proteins that position calcium channels for efficient neurotransmitter release. The β anchoring and regulatory protein, BARP, is an abundant integral membrane glycoprotein in the brain. Previous studies have shown the connection of BARP with the Ca_V_β subunit (Fig. 3A; Béguin et al. 2014). The interaction between these proteins involves domains I and II of BARP and the SH3 domain of the Ca_V_β subunit. Domain II is the region of the protein that allows for to pull-down of Ca_V_β_2a_/β_2b_, Ca_V_β_3_, and Ca_V_β_4_ (Béguin et al. 2014). The physiological consequence of this interaction is a decrease in the activity of channels without affecting their expression on the cell surface.

Of note, phenotypes in BARP knockout mice mirror those seen in models of Ca_V_1.2 channel inactivation via genetic or pharmacological means (Nakao et al. 2015). BARP-/- mice, as negative regulators of Ca_V_ channel activity, were expected to show a behavioral phenotype opposite to that of mice with Ca_V_1.2 alterations. However, the results did not meet this expectation. This observation may arise from compensatory mechanisms, in which Ca_V_β subunits substitute for BARP by binding proteins including RIMs, Rad, Rem, Gem, and Bassoon, thereby maintaining regulation (Buraei and Yang 2010; Puckerin et al. 2018), as elaborated below (Fig. 3A). RGK proteins seem to play a long-standing and well-established role in regulating channel activity. The molecular mechanisms of this regulation process appeared early in evolution and have remained mostly unchanged, underscoring their significance in animal biology (Puhl et al. 2014).

It has been reported that Rab3-interacting molecule 1 (RIM1), a crucial component of the synapse active zone, directly interacts with Ca_V_ channels (Kiyonaka et al. 2007). This interaction occurs via the SH3-HOOK-GK domains of the Ca_V_β subunits, whereas in RIM1, the carboxyl terminus seems to be the region that mediates this interaction (Fig. 3A). It has been reported that the voltage dependence of channel inactivation is shifted toward hyperpolarizing values by the interaction between Ca_V_β and RIM1 (Kiyonaka et al. 2007; Weiss et al. 2011). Furthermore, inhibiting this interaction accelerates channel inactivation and decreases acetylcholine (ACh) release in pheochromocytoma PC12 cells (Kiyonaka et al. 2007). Additionally, a functional interaction between RIM1 and L-type channels through the Ca_V_β_3_ auxiliary subunit has been identified, playing a role in regulating insulin secretion (Gandini et al. 2011; Weiss et al. 2011). These results show that Ca_V_β interacts with RIM1 to organize the vesicle release machinery.

RGK proteins regulate and integrate a range of intracellular signaling pathways. Rad is a monomeric protein similar to Ras that binds to the Ca_V_β subunit and reduces the current through Ca_V_1.2 L-type channels (Fig. 3A; Finlin et al. 2003; Béguin et al. 2007). This interaction regulates the protein’s localization to the T-tubule membrane in cardiomyocytes and is mediated by the Rad core motif and the highly conserved C-terminus of the protein (Elmore et al. 2024). Rad is a cell membrane-associated inhibitory regulator whose activity is controlled by a “phosphoswitch.” When this phosphoswitch is activated, it reduces Rad’s binding to the Ca_V_β subunit, which in turn lessens Rad’s inhibition of the current through the channels (Liu et al. 2020; Papa et al. 2021, 2024). Dynamic modulation of the Rad-Ca_V_β interaction in response to stress signals is crucial for cardiac adaptation (Wang et al. 2010). By modifying channel activity through Rad and its interaction with the Ca_V_β subunit, this regulation may ultimately explain how adrenergic signaling can enhance cardiac contractility. Therefore, regulating the activity of Ca_V_1.2 channels in the heart depends critically on the location of Rad in the membrane and its level of phosphorylation.

Rem is proposed to function as a negative regulator of L-type channels, specifically Ca_V_1.2 and Ca_V_1.3, by interacting with the Ca_V_β subunit. High glucose levels significantly increase the expression of Rem2 in pancreatic β cells, and its overexpression inhibits the function of Ca_V_ channels and the production of insulin. Although Rem2 interacts with several Ca_V_β subunits, it directly modifies the activity of the channels instead of influencing their trafficking to the membrane (Finlin et al. 2003; Chen et al. 2005; Leyris et al. 2009; Puckerin et al. 2016). It has also been suggested that Rem may inhibit channels through two distinct mechanisms using similar molecular determinants: one involves binding to the Ca_V_β subunit, and the other involves binding to the N-terminus of the Ca_V_1.2α_1_ subunit (Puckerin et al. 2016). Additionally, Rem2 has been reported to associate with the Ca_V_β_4_ subunit of Ca_V_2.1 neuronal Ca_V_ channels via low-affinity interactions (Xu et al. 2015).

This interaction, which presumably occurs through the GK domain in Ca_V_β, at least in Ca_V_β_3_, is independent of the current increase induced by the auxiliary subunit, and is crucial for Rem to inhibit channel activity (Béguin et al. 2007; Leyris et al. 2009). This interaction allows for precise and reversible inhibition of calcium current. It has also been emphasized that Rem’s localization to the plasma membrane is essential for its effective inhibition of L-type channels. Likewise, without membrane anchoring, Rem cannot effectively inhibit channel activity. Thus, one of the most important steps in the functional control of the channels is to direct Rem to the plasma membrane (Correll et al. 2007).

On the other hand, there is experimental evidence that Gem interacts with the Ca_V_β subunit to exert an inhibitory effect on Ca_V_ channels (Fig. 3A). It has been suggested that Gem’s ability to inhibit the channels depends on how the channel inactivates. Gem’s inhibition becomes stronger when the channel is inactivated. Gem keeps Ca_V_2.1 channels in their inactive state longer, which slows down their ability to open again and let calcium ions through (Allam et al. 2023).

The inhibitory effect of Gem on Ca_V_ channels also depends on the contribution of the auxiliary subunit Ca_V_β; however, it appears to be more complex than for other RGK proteins. Although Gem may not require direct interaction with Ca_V_β to inhibit channel activity, its presence seems to be essential for Gem to effectively conduct this inhibition. The auxiliary subunit allows the channel to form a restrictive site to which Gem binds to cause its inhibitory action. This process is known as the “Ca_V_β-priming” model (Fan et al. 2010, 2012).

Gem can affect Ca_V_2.3 channel functional expression through dynamin, a protein involved in endocytosis. This regulation apparently does not require a direct interaction between Ca_V_α_1_ and Ca_V_β, supporting the proposal of Ca_V_β priming in Gem-mediated Ca_V_2.3 channel regulation. Furthermore, the dynamin dependence suggests that Gem may decrease currents by promoting channel internalization or altering channel dynamics at the plasma membrane (Contreras et al. 2022).

Dynamin helps to endocytic vesicle formation and interacts with the Ca_V_β subunit through its SH3 domain (Fig. 3A; González-Gutiérrez et al. 2007; Miranda-Laferte et al. 2011). Remarkably, the loss of the intramolecular connection between the SH3 and GK domains inside Ca_V_β due to the association with dynamin may lead to subunit dimerization, a process suggested for Ca_V_ channel endocytosis (Miranda-Laferte et al. 2011). Furthermore, a peptide targeting the SH3 domain can block dynamin binding, thereby protecting Ca_V_1.2 channels from endocytosis. These data show that Ca_V_β regulates the channels’ membrane expression by means of dynamin-mediated endocytosis. Specifically, they suggest that the promotion of endocytosis and the effect of Ca_V_β on channel activation are mutually exclusive functions, regulated by subunit conformation, where dimerization may act as a functional switch that converts Ca_V_β from a channel activator to a promoter of its internalization (Miranda-Laferte et al. 2011).

The large synaptic protein Bassoon connects to the Ca_V_ channel complex by directly binding to the Ca_V_β subunit at presynaptic active zones (Fig. 3A; Carlson et al. 2010; Chen et al. 2011; Nishimune et al. 2012). Since the genetic deletion of Bassoon or the disruption of the interaction between Bassoon and the RIM-binding protein (RIM-BP) lowers synaptic levels of Ca_V_2.1, thereby impairing synaptic transmission, it has been proposed that this interaction may be direct or mediated by active zone proteins, such as RIM-BP (Davydova et al. 2014). The interaction between Bassoon and Ca_V_β affects synapse function. Bassoon acts as a scaffolding protein in the presynaptic zone, helping to position Ca_V_2.1 channels to neurotransmitter release sites through its interaction with RIM-BP. When Bassoon is deleted, or this connection is disrupted, the number of channels at the synapse decreases, impairing neurotransmitter release (He et al. 2018; Carlson et al. 2010).

On the other hand, it has been shown that the interaction between Bassoon with the Ca_V_β subunit, particularly linked with Ca_V_2.1 channels, modifies the voltage dependence of inactivation. As a result, the channels remain open longer during repetitive depolarization, which increases calcium influx and improves the efficiency of synaptic transmission at neuromuscular junctions (Nishimune et al. 2012).

The Ca_V_β subunit can be experimentally linked to Nedd4L, an E3 ubiquitin ligase, through engineered interactions (Fig. 3A). Specifically, fusing Nedd4L’s catalytic HECT domain to a nanobody (nb.F3) that targets Ca_V_β creates the chimeric nb.F3-Nedd4L (Ca_V_-aβlator), which selectively ubiquitinates Ca_V_β, reducing channel surface expression and currents without affecting total protein levels (Morgenstern et al. 2019). This experimental recruitment—rather than a native direct interaction—enables targeted posttranslational inhibition, disrupting Ca_V_β_1_-channel complex stability, open probability, and inactivation kinetics. Similarly, the Nedd4L HECT domain is fused to a Ca_V_β_1_-specific nanobody in Chisel-1, resulting in genetic suppression of Ca_V_β_1_-associated currents across heterologous and native systems (Morgenstern et al. 2022).

Ca_V_β subunits may interact with scaffolding proteins, influencing cytoskeletal dynamics. Ahnak is a scaffolding molecule and signaling protein. The interaction of this protein with the Ca_V_β subunits in various tissues has been described (Fig. 3A; Haase et al. 1999; Alvarez et al. 2004; Hohaus et al. 2002; Matza et al. 2008; Shao et al. 2009). The region in the auxiliary subunit that binds to Ahnak remains undetermined; what is known, however, is that the C-terminal domain of Ahnak participates in binding to the Ca_V_β_1_, Ca_V_β_2a_, and Ca_V_β_3_ subunits. Ahnak binds Ca_V_β_2a_ anchoring channels to the cytoskeleton and influences current amplitude/inactivation in cardiomyocytes and osteoblasts (Shao et al. 2009; Bünemann et al. 1999; Haase et al. 1999; Pankonien et al. 2011). PKA phosphorylation of Ca_V_β_2a_ (e.g., at sites like Ser-296; Pankonien et al. 2012) enhances its affinity for Ahnak, relieving Ahnak’s basal inhibitory effect on currents, rather than Ahnak driving phosphorylation. The interaction facilitates PKA regulation: phosphorylation disrupts Ahnak inhibition, increasing currents, but causality flows from PKA to Ahnak-Ca_V_β binding, not vice versa. No direct evidence shows Ahnak as a kinase scaffold or promoter of Ca_V_β_2a_ phosphorylation.

Another scaffolding protein that may bind the SH3 and GK domains of the Ca_V_β_2_ subunit is actin (Fig. 3A). In cardiomyocytes, Ca_V_β_2_ increases the activity of L-type channels, but blocking actin polymerization eliminates this effect (Stölting et al. 2015). Hidalgo and colleagues recently described the molecular details of how actin interacts with the Ca_V_β_2_ and Ca_V_β_4_ subunits of L-type channels (Castilla et al. 2025). These studies identified key regions in the Ca_V_β sequence required for its binding to actin. Mutations in these regions lowered Ca_V_β’s binding to actin and reduced current amplitude primarily due to more non-functional (conduction-silent) channels accumulating in the plasma membrane (Castilla et al. 2025).

## Interaction with Other Ion Channels and Receptors

Ca_V_β subunits may interact with other ion channels. The best-described association occurs with the skeletal muscle’s ryanodine receptor type 1 (RyR1). In this case, L-type channels interact with the RyR1s in the T-tubules to regulate the mechanism known as excitation-contraction (EC) coupling (Schneider and Chandler 1973; Rios and Brum, 1987) that triggers muscle contraction. The Ca_V_β_1a_ isoform seems to be essential for the functional organization of L-type channels coupled to RyR1s (Fig. 3A; Ahern et al. 2003; Buraei and Yang 2010). Charged residues are essential for the interaction in the region comprising aa M3201 and W3661, which has been identified as the Ca_V_β_1a_ binding site on RyR1s (Cheng et al. 2005). Mutation or neutralization of these residues disrupts RyR1-dependent calcium release during EC coupling. The Ca_V_β_1a_-RyR1 interaction is required for orthograde signaling, i.e., the transmission of the membrane depolarization signal detected by the L-type channel to the RyR1 for calcium release from the sarcoplasmic reticulum.

In cardiac muscle, the interaction between Ca_V_β and ryanodine receptors (RyRs) is not well defined but is known to involve the Ca_V_β_2_ subunit and RyR type 2 (RyR2). Both proteins work together to regulate calcium signaling for contraction, though the spatial organization of the complex is less regular than in skeletal muscle. It is important to note that in cardiac myocytes, clusters of Ca_V_1.2 channels, which include the Ca_V_β_2_ subunit, are located just nanometers away from RyR2 clusters within specialized junctions known as dyads or calcium release units. Most evidence suggests that Ca_V_β and RyR2s are close in function and space within dyadic nanodomains, but they may not interact directly or only with each other (Dixon 2022).

Petrovic et al. (2008) describe a cardiac complex linking Ca_V_1.2 (with Ca_V_β) to RyR2 via structural proteins, where PKA coordinates release (RyR2 phosphorylation) and entry, but Ca_V_β phosphorylation contributes little to current modulation compared to Rad. Likewise, these authors also characterized a dyadic complex linking Ca_V_1.2/Ca_V_β to RyR2 via intermediary proteins, in which PKA regulates RyR2 to mediate Ca²⁺ release and preferentially targets Rad (rather than Ca_V_β) to augment Ca_V_1.2 currents, enabling efficient cardiac excitation-contraction coupling. Ca_V_β’s phosphorylation contributes marginally to this process (Petrovic et al. 2008).

Bestrophin-1 (Best1) is a calcium-activated anion channel mainly located in the retinal pigment epithelium, where it may affect specific functional features of L-type channels, including their voltage-dependent activation and the strength of their currents (Rosenthal et al. 2006; Yu et al. 2008). In this case, the complex found in the plasma membrane is formed by Ca_V_1.3α1, Ca_V_β_3_ or Ca_V_β_4_, and Best1(Fig. 3A). Best1 engages the Ca_V_β SH3 domain via a proline-rich motif (aa 330–346) (Reichhart et al. 2010; Yu et al. 2008), while a novel C-terminal proline-rich motif (aa 468–486) is additionally essential for modulating Ca_V_β functions (Milenkovic et al. 2011).

It is noteworthy that Best1 channels may allow GABA to pass through and can interact with the GAD65 enzyme to enhance its activity (Lee et al. 2010; Oh and Lee 2017; Vargas-Parada et al. 2021). Although Best1 alone conducts GABA with a low relative permeability compared to chloride ions (0.09:1), GAD65 may increase Best1 permeability not only to GABA but also to glutamate (Wang et al. 2024). Furthermore, GABA can bind to an extracellular site on Best1 and stimulate chloride currents even at low concentrations, indicating that Best1 functions as a GABA-activated chloride channel, rather than serving primarily as a GABA-permeable channel on its own (Pant et al. 2025). Although the interaction between the Ca_V_β subunits and Best1 channels could be important for the correct membrane localization and functional regulation of Ca_V_ channels (Milenkovic et al. 2011), the precise function of this interaction in the Best1 channel’s functional characteristics is yet unknown.

Likewise, large-conductance calcium-activated potassium (BK) channels are found in excitable and non-excitable cells. As its name indicates, these channels are activated by membrane depolarization and the binding of cytosolic calcium (Sancho and Kyle, 2021). While Ca_V_β-BK interactions are not firmly established yet, it has been proposed that Ca_V_β_1_ binds Slo1 through its SH3 domain and the potassium channel’s calcium bowl region (Zou et al. 2008; Fig. 3B, upper panel). Slo1 interacts directly with the Ca_V_β_1_ subunit, independent of other proteins, strongly altering BKCa channel gating. This complex slows voltage-activated BKCa opening and lowers apparent calcium sensitivity, inhibiting gating at moderate cytoplasmic levels of the ion. These effects occur in heterologous systems without other Ca_V_ subunits and don’t notably change Slo1 surface expression, per electrophysiology and biochemistry (Zou et al. 2008). However, Ca_V_β_1_ increases membrane expression of Ca_V_ channels, augmenting depolarization-triggered calcium influx. It also slows Ca_V_ activation kinetics and inhibits BKCa channels, weakening their role as the main negative feedback for this influx. This does not saturate physiologically, since overexpression in ciliary ganglion neurons slowed kinetics, reduced macroscopic calcium-activated K⁺ currents, and raised L-type Ca²⁺ currents (Berkefeld et al. 2006; Loane et al. 2007).

The Ca_V_β_3_ subunit attaches to the inositol 3-phosphate (IP3) receptor in fibroblasts and pancreatic cells, making them less sensitive to IP3 and changing how calcium is released from the ER (Berggren et al. 2004; Belkacemi et al. 2018; Becker et al. 2021; Martus et al. 2024). This process limits glucose-stimulated insulin release in pancreatic β cells and decreases fibroblast motility by restricting calcium release from internal stores (Becker et al. 2021; Belkacemi et al. 2018; Berggren et al. 2004). These findings demonstrate the impact of the Ca_V_β_3_ subunit on free cytosolic calcium levels.

## Ca_V_β Subunits in the Cell Nucleus: Role in Transcription Control

Various proteins involved in nuclear signaling pathways that regulate key cellular processes such as survival, differentiation, and proliferation can also interact with Ca_V_β. Research indicates that the Ca_V_β subunits influence gene expression by binding to nuclear receptors and transcription factors, thereby modulating responses to hormones or small molecules. Nuclear localization has been documented for all Ca_V_β subunits. Proteome analysis has revealed its link to different proteins, as well as its participation in several nuclear functions.

Although the interactions and functions of Ca_V_β outside the channel complex have been studied in recent years, uncertainty persists regarding the mechanisms involved in its transport to the nucleus. Subramanyam and colleagues (2009) initially suggested that the N-terminus might control the nuclear localization of Ca_V_β subunits. To test this, they attached the N-terminus of Ca_V_β_4_ to Ca_V_β_2_, which was thought to mainly be found at the plasma membrane. In this study, the authors proposed that the N-terminal double arginine motif of Ca_V_β_4_ might enable nuclear targeting, an attribute that could be transferred to the Ca_V_β_2_ subunit, indicating isoform-specific mechanisms for nuclear translocation. More recently, Miranda-Laferte et al. (2025) demonstrated that the Ca_V_β_2_ subunit translocates independently to the nucleus, where it regulates transcription.

**Fig. 4:**
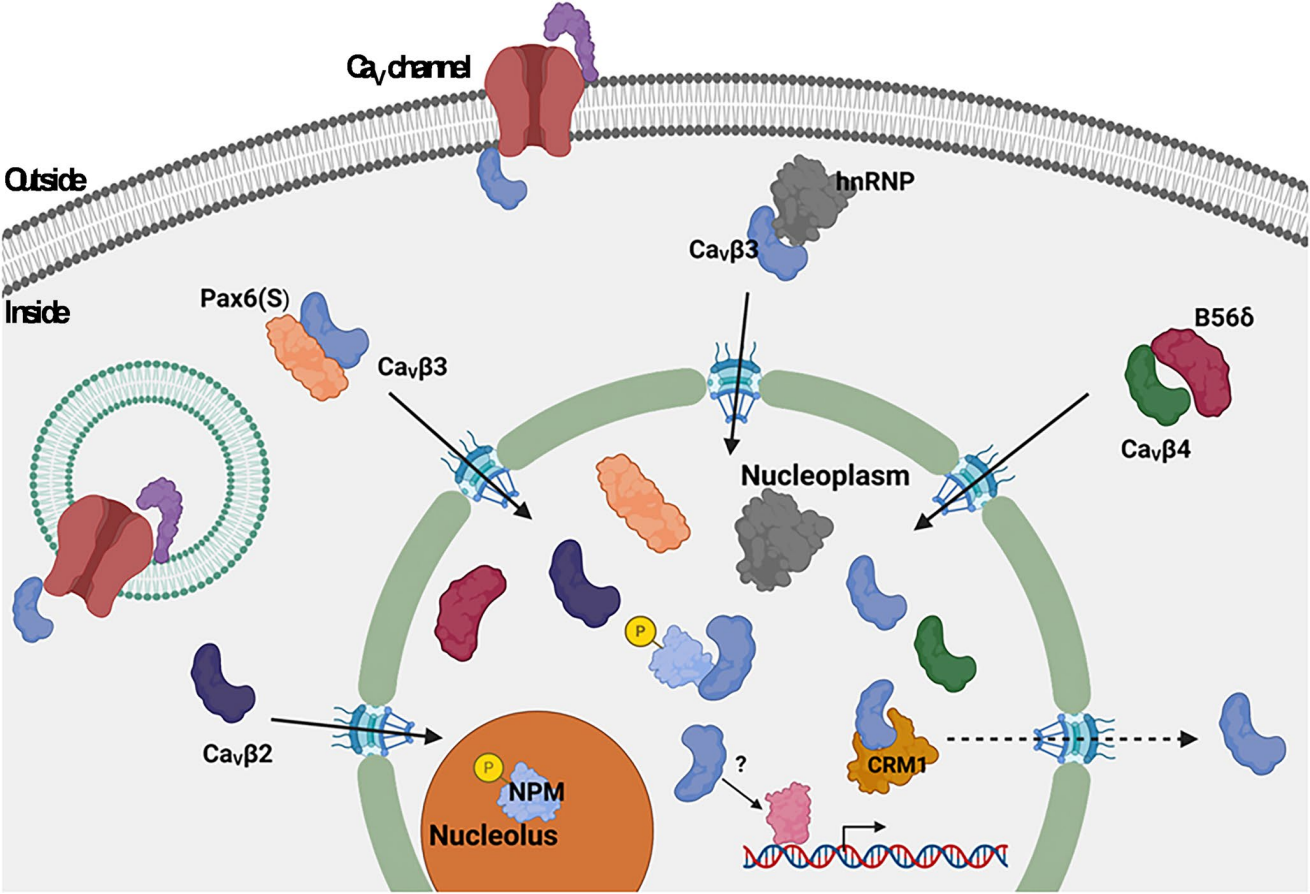
Nuclear transport of Ca_V_β subunits. A) Ca_V_β subunits can translocate to the nucleus and regulate gene expression. However, the NLS of Ca_V_β_3_ is not functional on its own, but instead enters the nucleus via a “piggyback” mechanism, binding to other proteins, such as hnRNPs or B56δ, just as subunit Ca_V_β_4_ does. In contrast, Ca_V_β_2e_ contains active nuclear localization signals at its N-terminus that directly mediate its nuclear targeting. The Ca_V_β_2e_ and Ca_V_β_3_ subunits contain nuclear export signals that allow them to move between the nucleus and cytoplasm in response to cellular signals

Even though studies suggests that a double-arginine motif in the N-terminal region of the Ca_V_β_4_ subunit isoform (aa 28–38) that might function as the NLS, it has been also found that the C-terminal region enables interaction with various proteins and facilitates transport of Ca_V_β to the nucleus. Earlier research indicates that the C-terminal truncated form of Ca_V_β_4_, known as R482X, does not translocate to the nucleus. This is likely because it cannot bind to B56δ, a regulatory subunit of protein phosphatase 2 A (PP2A; Tadmouri et al. 2012). Similarly, Ca_V_β_3_ may translocate to the nucleus via a piggyback mechanism mediated by proteins like B56δ and heterogeneous nuclear ribonucleoproteins (hnRNP), since its intrinsic nuclear localization signals (NLS) seem to be inactive (Fig. 4A; Corzo-Lopez et al. 2023).

In addition, it has been reported that Pax6(S), a short isoform of the full-length protein, may allow Ca_V_β_3_ to be localized in the nucleus (Fig. 4; Zhang et al. 2010) and function as a transcriptional regulator since its expression suppresses the transcriptional activity of Pax6(S). The hnRNP family, particularly the L and Q isoforms, represents another set of nuclear proteins that interact with Ca_V_β_3_ (Fig. 4). Thus, in addition to RNA biogenesis regulation, these proteins may facilitate nuclear transport of Ca_V_β_3_.

A functional nuclear export signal (NES) in Ca_V_β_3_ has been found to facilitate export from the nucleus, completing the protein’s nuclear transport cycle (Corzo-López et al. 2023). Similarly, it has recently been reported that the Ca_V_β_2e_ subunit can dynamically translocate from the plasma membrane to the nucleus. Nuclear import and export signals at its N-terminus regulate the nuclear translocation of Ca_V_β_2e_. Once inside the nucleus, the protein could control gene expression, acting also as a transcriptional regulator (Pickel et al. 2021; Miranda-Laferte et al. 2025). Moreover, silencing of Ca_V_β_2_ enhances cardiomyocyte hypertrophy triggered by α1-adrenergic receptor agonists. Nuclear Ca_V_β_2_ levels decline during hypertrophy progression; conversely, elevated levels inhibit hypertrophy, indicating a protective role in cardiomyocyte growth and function regulation (Pickel et al. 2021).

**Fig. 5:**
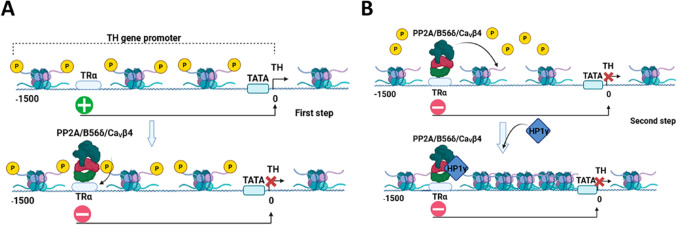
The Ca_V_β_4_ subunit facilitates gene transcription. (**A**) Following membrane depolarization, Ca_V_β_4_ forms a complex, Ca_V_β_4_/B56δ/PP2A, that interacts with the promoter region of specific genes, such as the TH gene, via nuclear transcription factors such as TRα. (**B**) The complex also binds to histones and influences chromatin structure, including the dephosphorylation of histone H3 through PP2A activity, resulting in the repression of gene expression. The HP1γ protein plays a key role in regulating gene transcription through a process facilitated by the Ca_V_β_4_ subunit. HP1γ helps regulate gene transcription by changing chromatin structure and interacting with transcription factors and chromatin-modifying complexes

In line with this, another known mechanism of nuclear localization and involvement in transcription for Ca_V_β subunits is the one described for the Ca_V_β_4_ variant, which involves its interaction with the regulatory component of PP2A, B56δ (Tadmouri et al. 2012; Ronjat et al. 2013). After translocating to the cell nucleus, the Ca_V_β_4_-B56δ/PP2A complex attaches to the thyroid hormone receptor α. This then enables association with the promoter of the tyrosine hydroxylase (TH) gene. Once associated with the promoter, this multiprotein complex binds to HP1γ, which dephosphorylates histone H3 at Ser10 through the action of PP2A, thereby repressing TH gene transcription (Tadmouri et al. 2012; Ronjat et al. 2013). The Ca_V_β_4_ subunit is translocated to the nucleus when neurons are electrically active, after its association with Ppp2r5d and PP2A, and this process, as mentioned above, is associated with the regulation of gene transcription (Fig. 5A; Ronjat et al. 2013). Mutations in Ca_V_β_4_ that abrogate nuclear translocation disrupt nuclear signaling pathways and are implicated in juvenile epilepsy (Etemad et al. 2014b).

Ca_V_β_4_ may regulate cellular proliferation by inducing cell cycle arrest at the G1 phase preceding S phase entry. This is likely linked to changes in the expression of the axin 2 and cyclin D genes when Ca_V_β_4_ is expressed. Indeed, it has been described that this subunit interacts with TCF4, a transcription factor that activates the Wnt/β-catenin pathway to control the expression of axin 2 and cyclin D (Rima et al. 2017a, b).

The Ca_V_β_4_ has also been found in the nucleus of hippocampal neurons during the early stages of development. The protein displays a nuclear localization pattern at least during the first 4 d in culture. However, when analyzing the subcellular distribution of the protein isoforms at division 21, it is observed that Ca_V_β_4a_-β_4b_ are located mainly in the cytoplasm. Interestingly, blocking electrical activity with TTX causes the Ca_V_β_4b_ isoform to relocate to the nucleus, while Ca_V_β_4a_ exhibits nuclear localization only to a limited extent. Once in the nucleus, both isoforms may help control genes that influence cell signaling, membrane transport, and nerve cell growth (Etemad et al. 2014a).

On the other hand, due to its interaction with HP1γ, a protein that binds to histones to silence gene transcription, the Ca_V_β_4c_ isoform has been associated with decreased gene expression. The physiological setting in which the Ca_V_β_4c_/HP1γ complex operates is unknown. However, it is known that a PXVXL motif at the protein’s C-terminal mediates its interaction by binding to the chromodomain of HP1γ (Fig. 5B; Hibino et al. 2003; Xu et al. 2011).

Lastly, Ca_V_β_1_ is the main variant expressed in skeletal muscle and is part of the Ca_V_1.1 channel complex in transverse tubules, supporting EC coupling. The embryonic Ca_V_β_1e_ isoform (in mouse, the “embryonic” stage spans from fertilization (E0) to ~ E15-E16, encompassing organogenesis and major morphogenesis) is located in cell nuclei and close to Z lines, while the postnatal Ca_V_β_1d_ isoform remains linked to the channels in the sarcolemma (Traoré et al. 2019). Additionally, it has been found that Ca_V_β_1a_ may also function as a transcriptional regulator during early myogenesis. This isoform can directly bind to the myogenin gene promoter at non-canonical E-box sites, repressing its transcription, affecting cell division, and favoring muscle progenitor cells to grow (Taylor et al. 2014). Therefore, one of the most important mechanisms for guaranteeing appropriate muscle progenitor cell expansion and muscle formation is the Ca_V_β_1a_ subunit’s inhibition of myogenin expression. Notably, Ca_V_β_1a_ is present in proliferating muscle progenitor cells and enters the nucleus before the expression of the Ca_V_1.1α_1_ ion-conducting subunit (Taylor et al. 2014).

## Non-canonical Interactions of Ca_V_α_2_δ: Its Role as a Synapse Organizer

The Ca_V_α_2_δ subunit has a complex structure with a heavily glycosylated α2 peptide outside the cell, a membrane anchor in the δ peptide, and several domains crucial for channel trafficking and regulation (Fig. 2B; Zamponi et al. 2015). This structure enables it to regulate Ca_V_ channel activity and membrane localization as well as to coordinate synaptic function. Indeed, the interactions that Ca_V_α_2_δ maintains with molecules outside the channel complex not only regulate the density and function of channels at the cell membrane but also affect other channels and receptors involved in cellular and synaptic processes beyond neurotransmission, as we shall discuss next.

The Ca_V_α_2_δ subunits share core structures but differ in length, motifs, and functions. Ca_V_α_2_δ−1 (~ 1,000 aa) and Ca_V_α_2_δ−2 (~ 1,100 aa) show 20–30% identity; this drops further with Ca_V_α_2_δ−3 (~ 1,000 aa) and Ca_V_α_2_δ−4 (~ 1,200 aa). These variants have unique glycosylation sites, variable post-cleavage linkers, and specific insertions in Cache domains. Ca_V_α_2_δ−3/4 subunits exhibit atypical MIDAS motifs deficient in key coordinating residues, which may impair cation binding and conformational changes. However, all Ca_V_α_2_δ proteins retain highly conserved topology as GPI-anchored membrane proteins, except Ca_V_α_2_δ−2 with its longer N-terminal signal sequence (Dolphin 2018).

## Molecular Interactions between the Ca_V_α_2_δ Subunit and Receptors and Channels

**Fig. 6:**
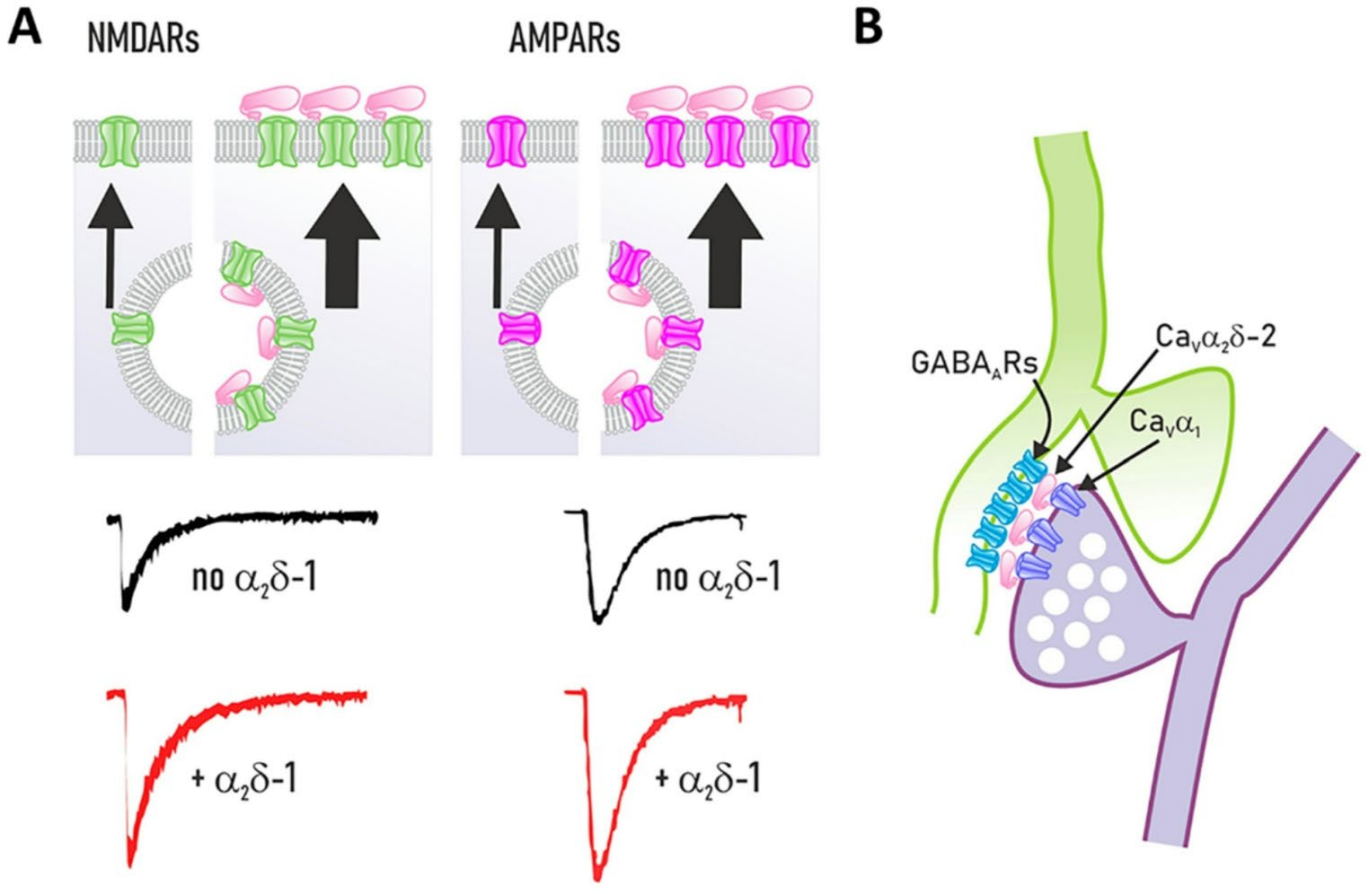
The CaVα2δ subunits regulate the expression and organization of neurotransmitter receptors. (**A**) CaVα2δ−1 associates with the NMDAR through its C-terminal, creating a complex that controls the synaptic trafficking and localization of the glutamate receptors, increasing their activity. This interaction is relevant in physiological processes such as synaptic plasticity and pathological processes such as neuropathic pain. Recent studies indicate that CaVα2δ−1 also interacts with the AMPA receptor and controls its composition in terms of its subunits and membrane expression. (**B**) Presynaptic overexpression of CaVα2δ−2 selectively increases clustering of postsynaptic GABAA receptors via a trans-synaptic mechanism, rather than through a direct physical binding interaction, which can cause aberrant synaptic wiring

New findings reveal that Ca_V_α_2_δ subunits from presynaptic channels interact directly or indirectly with postsynaptic neurotransmitter receptors and other ion channels at both pre- and postsynaptic sites. Specifically, the Ca_V_α_2_δ−1 subunit may serve as an auxiliary protein that forms a signaling complex with N-methyl-D-aspartate receptors (NMDARs; Fig. 6A). This interaction contributes to determining the trafficking, localization, and function of these neurotransmitter receptors at synapses (Chen et al. 2018; Zhou et al. 2021a, b). Furthermore, this molecular interaction reduces magnesium blockade of NMDAR channels, facilitating calcium entry and, therefore, enhancing their activity. Protein kinase C (PKC) phosphorylates NMDAR subunits at specific sites, S929 on GluN2A and S1413 on GluN2B, allowing them to form a complex with Ca_V_α_2_δ−1 (Zhou et al. 2021a).

The Ca_V_α_2_δ−1-NMDAR complex is a dynamic and regulated entity, influenced by neuronal activity and phosphorylation events, which constitute important mechanisms for synaptic plasticity. The molecular mechanisms underlying synaptic plasticity, which is essential for learning and memory, underscore the significance of Ca_V_α_2_δ−1-NMDAR complexes. These protein complexes in the dorsomedial striatum are critical for increasing glutamatergic activity in medium spiny neurons, which are responsible for synaptic modifications during long-term potentiation (LTP). Mice lacking the Ca_V_α_2_δ−1 subunit exhibit difficulties in learning and memory tasks, indicating deficits in cognitive function (Zhou et al. 2018b).

This interaction plays a key role in diseases such as neurogenic hypertension, neuropathic pain, and stroke, where increased NMDAR activity at synapses makes the condition worse (Chen et al. 2018; Luo et al. 2018; Wu et al. 2023). Moreover, the therapeutic effect of gabapentinoids is linked to their capacity to interfere with the interaction between Ca_V_α_2_δ−1 and NMDARs. Direct interaction between Ca_V_α_2_δ−1 and NMDARs may also occur in neurons of the spinal dorsal horn (Chen et al. 2018; Huang et al. 2020, 2024). Notably, overexpression of Ca_V_α_2_δ−1 caused by nerve injury increases presynaptic and postsynaptic NMDAR activity, resulting in pain hypersensitivity. Gabapentin (GBP) and peptides that interfere with the C-terminus of Ca_V_α_2_δ−1 can normalize the NMDAR targeting and synaptic activity that are increased after nerve injury, thereby providing a mechanism for the drug’s analgesic effects (Chen et al. 2018). Gabapentinoids relieve neuropathic pain by binding to Ca_V_α_2_δ−1 and disrupting the intracellular trafficking of the Ca_V_α_2_δ−1-NMDAR complex to the membrane, thereby decreasing synaptic NMDAR hyperactivity (Wu et al. 2023; Chen et al. 2018).

The predominant role of the Ca_V_α_2_δ−1-NMDAR complex in the pathogenesis of hypertension is mediated through its activity within the hypothalamus paraventricular nucleus. In this region, Ca_V_α_2_δ−1 interacts with NMDARs to increase excitatory glutamate signaling in presympathetic neurons. This causes an increase in sympathetic vasomotor tone and sympathetic outflow (Ma et al. 2018a, b; Zhou et al. 2021a; 2022). In conditions such as calcineurin inhibitor-induced and angiotensin II-associated hypertension, Ca_V_α_2_δ−1-NMDAR complexes enhance excitatory transmission (Zhou et al. 2023, 2025).

Experimental evidence further suggests an interaction between Ca_V_α_2_δ−1 and AMPA glutamate receptors (AMPARs; Fig. 6A). Studies have shown that α-Nrxn1α positively regulates calcium entry via Ca_V_2.1 channels that include Ca_V_α_2_δ−1. The Ca_V_α_2_δ−1 subunit connects Ca_V_ channels to AMPARs, affecting synaptic plasticity and pain pathways (Leana-Sandoval et al. 2024). This interaction modulates synaptic activity and plasticity, with the Ca_V_α_2_δ−1 subunit critically governing AMPAR intracellular trafficking, neuronal excitability, and synaptic functions essential for learning, memory, and pain processing (Leana-Sandoval et al. 2024).

In summary, these studies show that Ca_V_α_2_δ−1 amplifies spinal nociceptive input in neuropathic pain by binding phosphorylated NMDARs and GluA1/GluA2 AMPAR subunits via its C-terminal region (Huang et al. 2025). This interaction boosts synaptic NMDAR targeting, disrupts AMPAR assembly, and elevates presynaptic/post-synaptic calcium—making Ca_V_α_2_δ−1 a key gabapentinoid target that reduces pain by inhibiting receptor activity. Additionally, Ca_V_α_2_δ−1 fine-tunes AMPAR localization to regulate spatial memory, LTP, and seizure susceptibility, positioning it as an extracellular AMPAR organizer (Leana-Sandoval et al. 2024).

Likewise, presynaptic overexpression of a splice isoform of the Ca_V_α_2_δ−2 subunit lacking exon 23 (α_2_δ−2_ΔE23) at inhibitory synapses has been reported to result in a significant increase in the abundance of postsynaptic GABAA receptors (Fig. 6B; GABAARs). Interestingly, the impact of presynaptic Ca_V_α_2_δ−2 is also seen at glutamatergic synapses, resulting from abnormal connectivity between presynaptic glutamatergic boutons and postsynaptic GABAergic sites (Geisler et al. 2019). However, the presynaptic Ca_V_α_2_δ−2 subunit has been shown to regulate postsynaptic GABAAR levels indirectly. Although this regulation apparently does not depend on any of the known cell adhesion molecules, it could be strongly influenced by Nrxn1α (Geisler et al. 2019).

This is apparently similar for the Ca_V_α_2_δ−1 subunit, which also has a splice isoform lacking exon 23 (Ca_V_α_2_δ−1_ΔE23), but not in Ca_V_α_2_δ−3, where splicing apparently does not occur (Haddad et al. 2024). This was demonstrated when studying whether presynaptic expression of Ca_V_α_2_δ−1_ΔE23 altered trans-synaptic signaling in hippocampal neurons. To do this, neurons were transfected, and markers of glutamatergic (vGLUT1) and GABAergic (GABAARs) synapses were analyzed. In typical neurons, presynaptic glutamatergic boutons and postsynaptic GABAAR receptors remain separate. Yet, in neurons expressing the Ca_V_α_2_δ−1_ΔE23 splice isoform, these boutons and receptors overlap, suggesting abnormal signaling (Haddad et al. 2024). Therefore, beyond their traditional role in calcium channel regulation, Ca_V_α_2_δ subunits act as transsynaptic organizers that affect synapse specificity, postsynaptic receptor localization, and neuronal wiring.

A homozygous p.R593P mutation in Ca_V_α_2_δ−2 has recently been shown to impair channel function and synaptic activity. The mutation reduces the amount of the Ca_V_α_2_δ−2 protein on the cell membrane and its targeting to synapses. This affects the functioning of postsynaptic Ca_V_1.3 channels and reduces the number and activity of presynaptic Ca_V_2.1 channels. As a result, calcium signaling is impaired, which disrupts the normal communication between neurons. The mutation also causes fewer GABAA receptors to be recruited at synapses and reduces clustering of synapsin at excitatory glutamatergic synapses (Haddad et al. 2025).

**Fig. 7:**
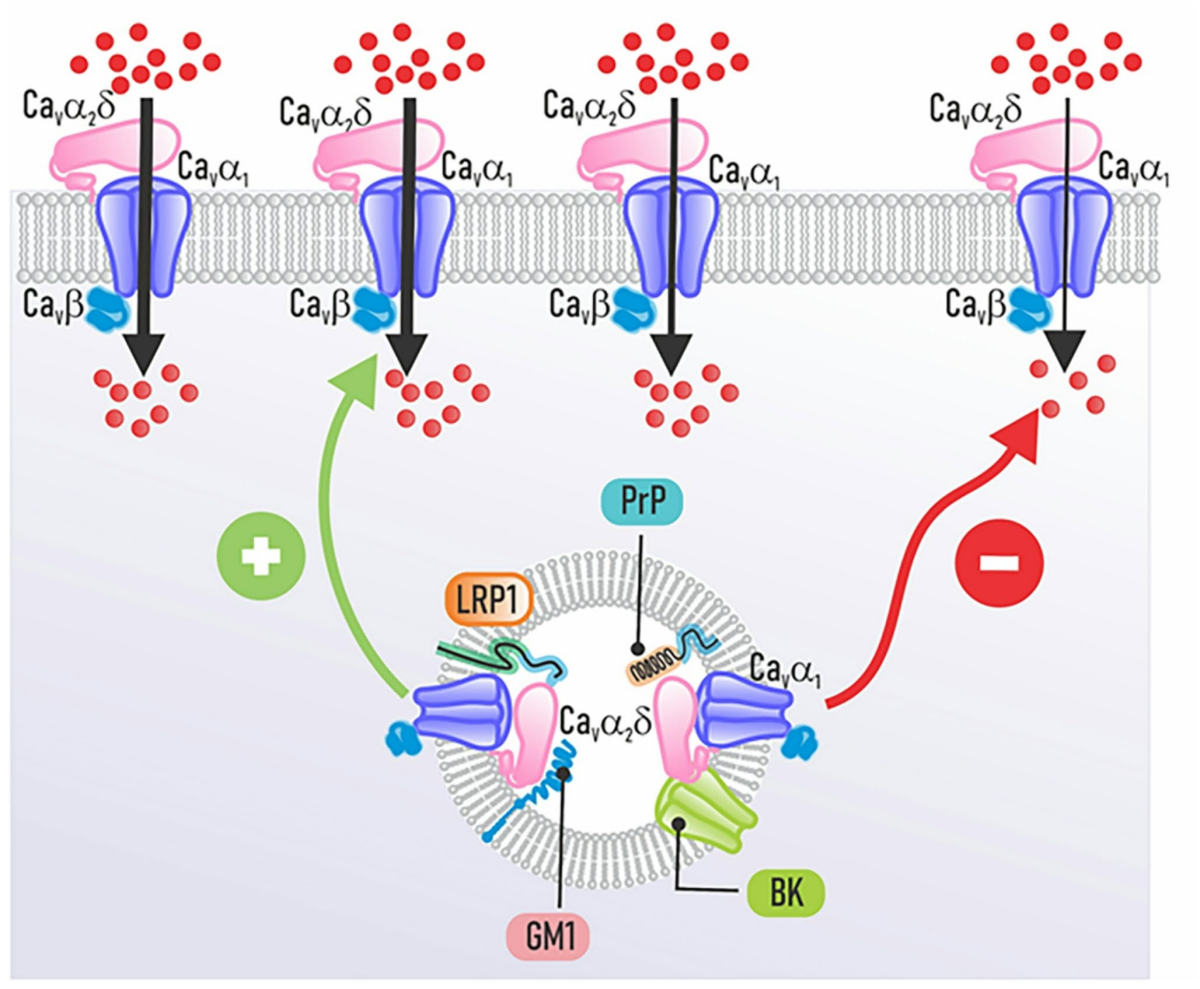
Broadening perspectives on Ca_V_α_2_δ subunit interactions beyond the Ca_V_ channel complex. Ca_V_α_2_δ directly interacts with membrane and extracellular molecules that regulate the A) channel complex. Interactions with prion proteins (PrP, normal brain proteins) and BK channels inhibit channel trafficking to the cell surface. It also binds LRP1 and ganglioside GM1 (a membrane glycosphingolipid), increasing the number of channels in the membrane

On the other hand, there is experimental evidence for a biochemical and functional interaction between Ca_V_α_2_δ subunits and BK channels, which mediate the reciprocal regulation of intracellular channel trafficking and cell electrical activity. Co-expression of BK channels with Ca_V_2 channels reduces their cell surface expression and current density by competing for the Ca_V_α_2_δ subunit (Fig. 7), thereby modulating the functional expression of Ca_V_ channels. In this regard, the work of Loane and colleagues (2007) initially documented that BK and Ca_V_2.2 channels physically and functionally coassemble in hippocampal pyramidal neurons, allowing rapid coupling where calcium influx activates BK channels to facilitate action potential repolarization. A study using recombinant channels without the Ca_V_α_2_δ subunit unexpectedly found rapid functional coupling similar to that in neurons, initially suggesting that this interaction might occur without the subunit in that experimental system (Loane et al. 2007). However, subsequent studies indicate that Ca_V_-BK channel interactions require the presence of Ca_V_α_2_δ, although direct binding between CaVα1 and BK subunits cannot be ruled out (Zhang et al. 2018; Shah et al. 2022; Gandini and Zamponi 2022).

The binding of the BK channel’s extracellular N-terminal domain to the Ca_V_α_2_δ subunit controls how calcium channels are transported within the cell and how they function, affecting their assembly and stability on the cell surface (Zhang et al. 2018; Shah et al. 2022). This interaction strengthens the tight spatial coupling (nanodomain) between calcium influx and BK channel activation, allowing for precise and rapid responses to calcium signals (Gandini and Zamponi 2022; Shah et al. 2022; Dolphin 2018). Thus, BK channels act as a feedback system to control calcium entry by helping the cell membrane return to its resting state after calcium enters. But when BK and Ca_V_2.2 channels are both present, they might compete for the Ca_V_α_2_δ subunit, which can reduce calcium flow through these channels. This interaction affects synaptic transmission by modifying the long-term expression and function of Ca_V_ channels in the cell membrane (Zhang et al. 2018). Furthermore, post-translational modifications (phosphorylation and palmitoylation) of BK channels modulate their binding affinity to the Ca_V_α_2_δ subunit, facilitating dynamic regulation of their interaction (Zhang et al. 2018). This interaction may help relieve pain by affecting how pain signals are controlled.

## Ca_V_α_2_δ−1 Subunit Interaction with Extracellular Matrix Proteins

The first experimental evidence of the interaction of the Ca_V_α_2_δ−1 subunit with a protein outside the channel complex corresponds to TSPs, a family of proteins secreted by astrocytes that promote synaptogenesis (Eroglu et al. 2009). These proteins serve as Ca_V_α_2_δ−1 extracellular ligands. The interaction occurs when the VWF-A domain of Ca_V_α_2_δ−1 binds to epidermal growth factor-like repeats of TSPs. This interaction is essential for the formation of excitatory synapses both in vitro and in vivo. GBP opposes this binding and thus prevents synaptogenesis (Eroglu et al. 2009).

The binding of Ca_V_α_2_δ−1 to TSPs causes structural modifications that activate a synaptogenic signaling complex. This attracts proteins and molecules needed to form new synapses (Eroglu et al. 2009). Building on these findings, Ca_V_α_2_δ−1 has been established as a crucial molecular mediator between astrocytes and neurons in regulating synaptogenesis through TSPs. Of the four Ca_V_α_2_δ variants, only Ca_V_α_2_δ−1 is related to TSPs, particularly TSP-1, TSP-2, and TSP-4 (Dolphin 2018). Consistent with this, inactivation of Ca_V_α_2_δ−1 expression results in significant alterations in excitatory synapse number, synaptic ultrastructure, synaptic activity, and a reduction in spinogenesis in mice.

Furthermore, the interaction between TSP and Ca_V_α_2_δ−1 has been described to control synaptogenesis through the small Rho GTPase Rac1 (Risher et al. 2018). Experimental Rac1 inhibition mimics the synaptic and spinal defects caused by the loss of the Ca_V_α_2_δ−1 subunit, whereas Rac1 activation can repair spinogenesis but not synapse formation. Therefore, it has been proposed that Ca_V_α_2_δ−1 might coordinate synapse and spine formation by coupling extracellular thrombospondin signals with intracellular Rac1 activation and cytoskeletal remodeling (Risher et al. 2018). Thus, a model was originally proposed in which “a transmembrane domain and a cytoplasmic tail” of Ca_V_α_2_δ−1 might recruit guanine nucleotide exchange factors that in turn could activate Rac1 at the synapse (Risher et al. 2018). However, this model is questioned because the current topological model of Ca_V_α_2_δ−1 subunits shows they are fully extracellular proteins attached to the membrane via a GPI anchor (Davies et al. 2010), leaving the exact way Rac1 is activated unclear. These studies indicate that Ca_V_α_2_δ−1 contributes to synapse organization by interacting with TSPs and possibly involving the Rac1 signaling pathway, which may affect brain development and disease.

It is worth mentioning also that TSP-4 binds to the Ca_V_α_2_δ−1 subunit, causing a decrease in the binding affinity of GBP to the subunit (Lana et al. 2016). This effect is eliminated when mutations (D122A, D224A, S125A) are introduced in the VWF-A domain (Cantí et al. 2005). Additionally, the synaptogenic EGF-like domain of TSP-4 alone does not influence GBP binding affinity. TSP-4 and Ca_V_α_2_δ−1 interact inside the cell, and no obvious association between them is observed at the cell membrane (Lana et al. 2016). Following nerve injury, TSP-4 levels increase, altering calcium signaling within primary sensory neurons (Guo et al. 2017). When TSP-4 binds to Ca_V_α_2_δ−1, which is also overexpressed in neuropathic pain models, it alters calcium currents and also affects GBP binding (Guo et al. 2017). Interestingly, GBP does not disrupt the interaction between the Ca_V_α_2_δ−1 subunit and TSP-4 because it does not block their molecular binding.

A homozygous p.R593P mutation in Ca_V_α_2_δ−2 recently reported disrupts Ca_V_ channel function and synaptic activity. Presynaptic Ca²⁺ influx and synaptic transmission are potentiated by Ca_V_α_2_δ−1 interaction with Neurexin-1α (Nrxn1α), likely through Ca_V_2.1 modulation. Nrxn1α-deficient neurons show reduced presynaptic Ca²⁺ entry, impaired vesicle release, fewer synaptic Ca_V_2.1 channels, and increased axonal Ca_V_α_2_δ−1 trafficking (Brockhaus et al. 2018). It is important to highlight that overexpression of this protein restores calcium entry into the presynapse and, with it, the normal release of vesicles. Coexpression of Nrxn1α with the Ca_V_α_2_δ−1 subunit increases calcium currents, an effect that appears to be specific since it is not seen with the Ca_V_α_2_δ−3 variant (Brockhaus et al. 2018). Electrophysiological studies have shown that Nrxn1α increases calcium currents through recombinant Ca_V_2.1 channels containing the Ca_V_α_2_δ−1 subunit without significantly modifying their basic biophysical properties. These results suggest that Nrxn1α participates in the regulation of synaptic signaling through a direct interaction with Ca_V_α_2_δ−1, modulating it and probably contributing to determining the number of channels in the cell membrane. Likewise, pregabalin (PGB) is another high-affinity ligand that selectively binds to the Ca_V_α_2_δ−1 subunit, which has been reported to exert presynaptic inhibitory effects by acting on the protein to disrupt the functional coupling between Ca_V_ channels and synaptic vesicles. This mechanism is also affected by the interaction between PGB and Nrxn1α. PGB binding reduces the effective size of the rapidly releasable pool of synaptic vesicles without significantly decreasing presynaptic calcium influx (Martínez San Segundo et al. 2020). Overexpression of Ca_V_α_2_δ−1 potentiates the effects of PGB and Nrxn1α on synaptic vesicle release. In contrast, its inhibition hinders synaptic transmission. These findings indicate that Ca_V_α_2_δ−1 functions as a regulatory platform for extracellular signaling. It integrates both exogenous regulation by PGB and endogenous modulation by Nrxn1α to control neurotransmitter release by altering the coupling between Ca_V_ channels and secretory vesicles (Martínez San Segundo et al. 2020).

With implications for synaptic control and neurodevelopmental disorders, including autism, a conserved mechanism has been hypothesized in which Nrxn1α binds to Ca_V_α_2_δ subunits of Ca_V_2 channels and mediates retrograde inhibition of neurotransmitter release (Tong et al. 2017). In the *C. elegans* neuromuscular junction, postsynaptic Nrxn1α interacts with presynaptic Ca_V_α_2_δ−1 (UNC-36) of Ca_V_2.2 channels (UNC-2), decreasing calcium channel abundance and function and thus inhibiting Ach release. Retrograde inhibition involves a soluble Nrxn1α ectodomain released from the postsynaptic membrane by the protease SUP-17/ADAM10 (Tong et al. 2017). Nrxn1α reduces synaptic signaling and regulates synaptic transmission in mammals by directly binding to Ca_V_α_2_δ−3 and decreasing calcium currents through Ca_V_2.2 channels. This effect is selective, as Nrxn1α does not influence channels containing the Ca_V_α_2_δ−1 or Ca_V_α_2_δ−2 subunits (Tong et al. 2017).

This trans-synaptic connection mediated by Nrxn1α and Ca_V_α_2_δ subunits regulates calcium influx and contributes to determining neurotransmitter release, establishing a functional link between the presynaptic physical machinery responsible for release and the postsynaptic receptors. Mutations in genes that encode Nrxn1α and Ca_V_ channel subunits, including Ca_V_α_2_δ−3, have been linked to a higher risk of developing autism spectrum disorder (ASD). This means the pathway involving the interaction between Nrxn1α and Ca_V_α_2_δ−3 may have an important role in how ASD develops at the molecular level. Moreover, this interaction may affect synaptic plasticity and the coordination of postsynaptic responses, which are relevant for efficient neuronal communication and optimal brain functioning (Tong et al. 2017).

## Other Interactions

Low-density lipoprotein receptor-related protein 1 (LRP1) participates in the trafficking and surface expression of the Ca_V_α_2_δ subunits and other proteins linked to the Ca_V_ channel complex. Indeed, Ca_V_α_2_δ has been reported to act as a ligand for LRP1 (Fig. 7). This interaction occurs between the VWF-A domain of the Ca_V_α_2_δ−1 subunit and the ligand-binding domains II and IV of LRP1. The chaperone-associated receptor (CAR), which enables correct folding and trafficking of both LRP1 and its ligands, regulates this process (Kadurin et al. 2017; Dahimene et al. 2018). Coimmunoprecipitation experiments confirmed that an LRP1 minireceptor construct (LRP1-m4) binds to Ca_V_α_2_δ, and this binding increases when the Receptor-Associated Protein (RAP) is present (Kadurin et al. 2017). Studies with surface plasmon resonance reveal a strong, calcium-dependent interaction between LRP1 and Ca_V_α_2_δ−1. Other research shows that without LRP1, the transport of Ca_V_α_2_δ−1 to the membrane is disrupted, impairing calcium signals and weakening vascular smooth muscle contraction (Au et al. 2018).

These findings emphasize the crucial role of LRP1 in regulating the intracellular trafficking of the Ca_V_α_2_δ subunit, which in turn profoundly influences the expression of Ca_V_ channels at the plasma membrane. Research has shown that LRP1 influences the trafficking of Ca_V_α_2_δ−1 by altering its processing and modification, ultimately determining its localization on the cell membrane. Corneal proteins extracted under physiological conditions were subjected to LRP1 affinity screening, and LRP1 ligand candidates were identified by mass spectrometry. Twenty-eight LRP1 ligand candidates were found, including the Ca_V_α_2_δ−1 subunit (Mogensen et al. 2022). This finding supports the idea that LRP1 and Ca_V_α_2_δ−1 interact to regulate Ca_V_ channel trafficking and function.

Likewise, experimental evidence shows that the ganglioside GM1 interacts with the Ca_V_α_2_δ−1 auxiliary subunit (Fig. 6A). Studies in sperm and *Xenopus* oocytes have shown that GM1 influences calcium influx through Ca_V_2.3 channels, and this effect relies on the presence of Ca_V_α_2_δ−1. The Ca_V_α_2_δ−1 subunit is known to be located in membrane lipid raft domains, which are rich in GM1. The interaction between these components changes the channel’s voltage sensitivity, affecting crucial physiological processes such as the sperm acrosomal reaction. These findings suggest a functional and potentially direct interaction between these molecules (Cohen et al. 2014).

Specifically, sterol efflux and local enrichment of the ganglioside GM1 in the sperm membrane have been found to trigger calcium influx through Ca_V_2.3 calcium channels. These channels are apparently required for the acrosome exocytosis crucial for fertilization. Both the lipid and sugar components of GM1, as well as the auxiliary Ca_V_α_2_δ−1 subunit of Ca_V_2.3 channels, are required for this regulatory effect. Thus, this interaction model may help explain how membrane lipids can regulate channel function and, in turn, control calcium-dependent cellular processes such as exocytosis (Cohen et al. 2014).

Interactions between prion proteins (PrP) and Ca_V_α_2_δ subunits have been demonstrated in both heterologous expression systems and neurons (Fig. 7). Senatore and colleagues (2012) initially demonstrated that PrP impairs glutamatergic neurotransmission in the cerebellum by disrupting the intracellular trafficking of Ca_V_ channels. Specifically, a misfolded mutant PrP, retained in the ER, can bind to Ca_V_α_2_δ−1. The auxiliary subunit Ca_V_α_2_δ−1 builds up inside the cell as a result of this interaction, blocking the channel complexes’ trafficking to the cell membrane. This results in reduced calcium entry into the presynaptic terminals of cerebellar granule neurons, leading to decreased glutamate release and impaired synaptic function. In genetic models of prion disease, synaptic dysfunction precedes neuronal loss and motor deficits, linking the intracellular accumulation of mutant PrP and Ca_V_α_2_δ−1 to the early neurotoxic events in the disease (Senatore et al. 2012).

When PrP is expressed alongside channels that include Ca_V_2.1, Ca_V_β, and either Ca_V_α_2_δ−1 or Ca_V_α_2_δ−2 subunits, it leads to a decrease in the amount of calcium influx. This reduction requires the anchoring of PrP to the membrane via its GPI anchor. PrP without this anchor does not inhibit calcium currents. Functional assays have revealed that PrP likely interferes with Ca_V_α_2_δ function by competing for or disrupting GPI anchor-mediated localization. This implies that they might interact in membrane microdomains that are rich in cholesterol (Davies et al. 2010; Alvarez-Laviada et al. 2014). Overexpression studies indicate that PrP and Ca_V_α_2_δ can interfere with each other’s intracellular trafficking (Dolphin, 2018). This is likely due to their competition for a limited supply of GPI anchors or their use of shared trafficking pathways, such as endosomal recycling.

On the other hand, treatment with the prion peptide (PrP 106–126) has been reported to elevate intracellular calcium levels in neurons, which subsequently leads to neuronal damage and cell death. In this case, it has been proposed that calcium entry occurs through L-type channels and leads to a reduction in AMP-activated protein kinase (AMPK) activity, which induces cell death by autophagy. Activation of AMPK through its agonist AICAR reduces neuronal toxicity and decreases autophagy caused by PrP 106–126, showing a protective effect on neurons (Moon and Park, 2020).

Depending on the subtype and interaction mode, PrP modulates Ca_V_ channels variably; prion-related neurotoxicity involves both inhibition and enhancement of calcium influx. Other studies demonstrate that recombinant PrP inhibits L-type channels, thereby reducing calcium influx in specific neuronal populations (Korte et al. 2003). Likewise, though early studies in PrP-deficient mice showed that the absence of PrP may alter intracellular calcium dynamics in cerebellar granule cells, by a mechanism independent of changes in channel function (Herms et al. 2000). These studies also indicate that the absence of the protein reduces calcium influx through L-type channels and suggest that normal PrP may enhance calcium influx through these channels (Fuhrmann et al. 2006).

Finally, recent experiments show that Rab11, a small protein involved in recycling within cells, directly controls Ca_V_ channels’ functional expression by interacting with the Ca_V_α_2_δ−1 subunit (Fig. 7). This interaction regulates the intracellular trafficking and activity of neuronal Ca_V_2.2 channels at the cell surface. Furthermore, these studies have shown that Ca_V_α_2_δ−1 recycles to the plasma membrane through a Rab11a-dependent pathway. Consequently, dominant-negative Rab11a mutants significantly reduce Ca_V_α_2_δ−1 trafficking to the plasma membrane, which therefore implies an impairment of the functional expression of channels at the membrane. Likewise, coexpression of a dominant-negative mutant of Rab11a reduces calcium currents through Ca_V_2.2 channels only when Ca_V_α_2_δ−1 is present, but not when Ca_V_α_2_δ−3 is expressed. Rab11 regulates the intracellular trafficking and recycling of Ca_V_ channels containing the Ca_V_α_2_δ−1 subunit, thereby controlling their plasma membrane density and functional activity (Meyer and Dolphin 2021).

In addition, there is evidence indicating a key role for Rab11 in Ca_V_α_2_δ−2 intracellular trafficking and establishing the effect of GBP as an effective blocker of this recycling pathway. GBP blocks the Ca_V_α_2_δ−2 subunit from recycling to the plasma membrane from Rab11-positive endosomes without affecting its internalization (Tran-Van-Minh et al. 2010). This reduces membrane expression of Ca_V_α_2_δ−2 and consequently decreases currents through Ca_V_ channels. Research shows that a mutated form of Rab11 prevents GBP from lowering the levels of Ca_V_α_2_δ−2 on the cell membrane and reducing the activity of Ca_V_ channels. This suggests that GBP affects Rab11’s role in recycling Ca_V_α_2_δ−2, which helps to explain how GBP controls channel activity and provides its therapeutic benefits (Tran-Van-Minh et al. 2010).

### Future Directions

Research into the functional roles of the Ca_V_β and Ca_V_α_2_δ auxiliary subunits of voltage-gated calcium channels is progressing rapidly. There is a wide range of opportunities, with increasing interest in analyzing how these Ca_V_ auxiliary subunits function beyond their traditional roles within the channel complex. Growing evidence highlights the need for further research to identify additional molecular partners that interact with these proteins beyond those already known. These potential interactions may involve proteins located in the cytosol or other cellular compartments, as well as lipids, small molecules, and nucleic acids; all of which can directly influence cellular processes or serve as molecular scaffolds or regulators of signaling pathways.

Innovative techniques such as super-resolution and cryo-electron microscopy, which allow atomic-level characterization of the dynamic conformations of the auxiliary subunits and their interactions with partner molecules beyond Ca_V_ channel complexes, may be essential for designing targeted drugs or small-molecule modulators. Research on post-translational modifications—including sumoylation, phosphorylation, and ubiquitination—together with emerging regulatory mechanisms, has gained considerable prominence. Studying these changes could reveal how they influence the location, stability, and function of the channel auxiliary subunits within cells, impacting key signaling pathways involved in vital processes such as neural plasticity and cardiac remodeling. Developing molecules that target the interactions of Ca_V_ auxiliary subunits beyond the channel complex offers promising potential for treating neurodegenerative diseases, cancer, and cardiovascular disorders, with the possibility of making a meaningful difference in human health and biotechnology.

To gain a deeper understanding of the Ca_V_ accessory subunits’ biological roles, it is essential to study how gene regulation, alternative splicing, and epigenetic mechanisms influence their functions beyond their role in the channel context. Using systems biology and integrative omics techniques, which combine proteomics, genomics, and interactomics to map the complex regulatory networks of Ca_V_ accessory subunits, will greatly enhance our understanding of their roles in complex cellular processes and help identify potential targets for novel therapeutic strategies.

## Supplementary Information

Below is the link to the electronic supplementary material.

Supplementary Material 1

Supplementary Material 2

## Data Availability

No new data were created or analyzed in this study. Data sharing does not apply to this article.
