# Supplementary material for: Molecular Partners of Voltage-Gated Calcium Channel β and α2δ Auxiliary Subunits: Roles in Channel Complex Regulation and Beyond
Source: J Membr Biol. 2026 Mar 16;259(1):11. doi: 10.1007/s00232-026-00371-w (PMC12992490; doi:10.1007/s00232-026-00371-w)
Supplement: Supplementary file 1 — Supplementary Material 1 [file 232_2026_371_MOESM1_ESM.pdf]

Supplemental Table 1. Classification of voltage-gated (Ca_V) channels.

Gene	Protein	Former name	Current	
**CACNA1S**	$Ca_V1.1$	α_{1S}	L-type	
**CACNA1C**	$Ca_V1.2$	α_{1C}	L-type	
**CACNA1D**	$Ca_V1.3$	α_{1D}	L-type	
**CACNA1F**	$Ca_V1.4$	α_{1F}	L-type	**HVA**
**CACNA1A**	$Ca_V2.1$	α_{1A}	P/Q-type	
**CACNA1B**	$Ca_V2.2$	α_{1B}	N-type	
**CACNA1E**	$Ca_V2.3$	α_{1E}	R-type	
**CACNA1G**	$Ca_V3.1$	α_{1G}	T-type	
**CACNA1H**	$Ca_V3.2$	α_{1H}	T-type	**LVA**
**CACNA1I**	$Ca_V3.3$	α_{1I}	T-type	
