# Supplementary material for: Molecular Partners of Voltage-Gated Calcium Channel β and α2δ Auxiliary Subunits: Roles in Channel Complex Regulation and Beyond
Source: J Membr Biol. 2026 Mar 16;259(1):11. doi: 10.1007/s00232-026-00371-w (PMC12992490; doi:10.1007/s00232-026-00371-w)
Supplement: Supplementary file 2 — Supplementary Material 2 [file 232_2026_371_MOESM2_ESM.pdf]

Abbreviations:

ACh: Acetylcholine.
ADAM10: A disintegrin and metalloprotease 10.
AICAR: 5-Aminoimidazole-4-carboxamide ribonucleotide.
AID: Alpha-interaction domain.
Ahnak: AHNAK Nucleoprotein.
AMPA: α-amino-3-hydroxy-5-methyl-4-isoxazolepropionic acid.
AMPARs: AMPA glutamate receptors.
AMPK: AMP-activated protein kinase.
ASD: Autism spectrum disorder.
B56δ: PP2A regulatory subunit B56δ.
BARP: β anchoring and regulatory protein.
Best1: Bestrophin-1.
BK: Large-conductance calcium-activated potassium.
CAR: Chaperone-associated receptor.
CaV: Voltage-gated calcium channels or subunits.
$CaV\alpha_1$: Pore-forming α_1 subunit of voltage-gated calcium channels.
$CaV\alpha_2\delta$: $\alpha_2\delta$ auxiliary subunit of voltage-gated calcium channels.
$Ca_V\beta$: β auxiliary subunit of voltage-gated calcium channels.
CRM-1: Chromosome region maintenance 1.
Cryo-EM: Cryo-electron microscopy.
EC: Excitation-contraction.
EMC: Endoplasmic Reticulum Membrane Protein Complex.
EGF-like: Epidermal growth factor-like.
ER: Endoplasmic Reticulum.
ERAD: ER-associated degradation.
$GABA_ARs$: $GABA_A$ receptors.
GAD65: Glutamic acid decarboxylase 65.
GBP: Gabapentin.
Gem: RGK family GTPase.
GK: Guanylate cyclase-like.
GluA1/GluA2: AMPA receptor subunits.
GluN1/GluN2A, GluN2B: NMDA receptor subunits.
GM1: Ganglioside GM1.
GPI: Glycophosphatidylinositol.
HECT: Homologous to E6-AP carboxyl terminus.
hnRNP: Heterogeneous nuclear ribonucleoprotein.
HOOK: HOOK region (linker between SH3 and GK domains in $Ca_V\beta$).
HP1γ: Heterochromatin protein 1 gamma.
HVA: High-voltage activated (calcium channels).

IP3: Inositol 3-phosphate.
IP3R: IP3 receptor.
kDa: Kilodalton.
L-type: L-type calcium channels (Ca_V1 subfamily).
LRP1: Low-density lipoprotein receptor-related protein 1.
LRP1-m4: LRP1 minireceptor construct.
LTP: Long-term potentiation.
LVA: Low-voltage activated (calcium channels).
MAGUK: Membrane-associated guanylate kinase.
MIDAS: Metal ion-dependent adhesion site.
N-type: N-type calcium channels ($Ca_V2.2$).
Nedd4L: Neural precursor cell expressed, developmentally down-regulated 4-like (also called NEDD4-2).
nb.F3-Nedd4L: Nanobody-targeted Nedd4L chimera.
NES: Nuclear export signal.
PC12: Pheochromocytoma PC12 cells.
NLS: Nuclear localization signal.
NMDARs: N-methyl-D-aspartate receptors.
Nrxn1α: Neurexin-1α.
pLDDT: predicted Local Distance Difference Test
PEST: Proline-glutamic acid-serine-threonine motif.
P/Q-type: P/Q-type calcium channels ($Ca_V2.1$).
p.R593P: Homozygous mutation in $Ca_V\alpha_2\delta$-2.
Pax6(S): Short isoform of Pax6.
PKA: Protein kinase A.
PKC: Protein kinase C.
PLC: Phospholipase C.
PP2A: Protein phosphatase 2A.
PrP: Prion protein.
PrP 106-126: Prion peptide.
PxxP: Proline-rich motif.
R482X: C-terminal truncated $Ca_V\beta_4$ variant.
R-type: R-type calcium channels ($Ca_V2.3$).
Rab11: Rab11 GTPase.
Rac1: Rho GTPase.
Rad: Ras-related small GTPase.
RAP: Receptor-Associated Protein (enhances LRP1-$Ca_V\alpha_2\delta$ binding).
Rem: Ras-related small GTPase (RGK family member, including Rem2).
RGK: Ras-like GTPase family (small GTPases like Rad, Rem, Rem2).
RIM1: Rab3-interacting molecule 1.
RIM-BP: RIM-binding protein.

RyR1: Ryanodine receptor type 1.
RyR2: Ryanodine receptor type 2.
RyRs: Ryanodine receptors.
SH3: Src homology 3.
Slo1: Pore-forming subunit of BK channels.
TSPs: Thrombospondins.
T-tubules: Transverse tubules.
T-type: T-type calcium channels (Ca_V3).
TH: Tyrosine hydroxylase.
TTX: Tetrodotoxin.
UNC-2: *C. elegans* $Ca_V2.2$ channel ortholog.
vGLUT1: Vesicular glutamate transporter 1.
VWF-A: von Willebrand factor A domain.
